# Comparison between ^18^F-FDG PET/CT and diffusion-weighted imaging in detection of invasive ductal breast carcinoma

**DOI:** 10.22038/AOJNMB.2023.70534.1493

**Published:** 2024

**Authors:** Aynur Ozen, Tarik Sayin, Ozan Kandemir, Ozgul Ekmekcioglu, Serdar Altınay, Eylem Bastug, Ali Muhammedoglu, Atilla Celik, Ramazan Albayrak

**Affiliations:** 1 Department of Nuclear Medicine, Istanbul Bagcılar Training and Research Hospital, University of Health Sciences, Istanbul, Turkey; 2 Department of Radiology, Istanbul Bagcılar Training and Research Hospital, University of Health Sciences, Istanbul, Turkey; 3 Department of Nuclear Medicine, Mugla Training and Research Hospital, Mugla Sıtkı Kocman University, Mugla, Turkey; 4 Department of Nuclear Medicine, Istanbul Sisli Hamidiye Etfal Training and Research Hospital, University of HealthSciences, Istanbul, Turkey; 5 Department of Pathology, Istanbul Bakırköy Training and Research Hospital, University of Health Sciences, Istanbul, Turkey; 6 Department of Pathology, Istanbul Bagcılar Training and Research Hospital, University of Health Sciences, Istanbul, Turkey; 7 Department of General Surgery, Istanbul Bagcılar Training and Research Hospital, University of Health Sciences, Istanbul, Turkey; 8 Department of Radiology, Istanbul Medeniyet University, Faculty of Medicine, Istanbul, Turkey

**Keywords:** Breast carcinoma, Positron emission tomography, Standardized maximal uptake, Apparent diffusion coefficient Diffusion magnetic resonance imaging

## Abstract

**Objective(s):**

Breast carcinoma is the most common type of cancer in females. This study aims to compare fluorine-18-fluorodeoxyglucose (^18^F-FDG) uptake pattern and apparent diffusion coefficient (ADC) value for the detection of the primary tumour and axillary metastases of invasive ductal breast carcinoma.

**Methods:**

This study included 40 breast carcinoma lesions taken from 39 patients. After staging by positron emission tomography-computed tomography (PET/CT) and diffusion-weighted magnetic resonance imaging (MRI), breast surgery with axillary lymph node dissection or sentinel lymph node biopsy was performed.

**Results:**

Primary lesion detection rate for PET/CT and diffusion-weighted MRI was high with 39 of 40 lesions (97.5%). The sensitivity and specificity for the detection of metastatic lymph nodes in axilla were 40.9%, 88.9%, with ^18^F-FDG PET/CT scans and 40.9%, 83.3%, for dw-MRI, respectively. No significant correlation was detected between ADC and SUV_max_ or SUV_max_ ratios. Estrogen receptor (p=0.007) and progesterone receptor (p=0.036) positive patients had lower ADC values. Tumour SUV_max_ was lower in T1 than T2 tumour size (p=0.027) and progesterone receptor-positive patients (p=0.029). Tumour/background SUV_max_ was lower in progesterone receptor-positive patients (p=0.004). Tumour/liver SUV_max_ was higher in grade III patients (p=0.035) and progesterone receptor negative status (p=0.043).

**Conclusions:**

This study confirmed the high detection rate of breast carcinoma in both modalities. They have same sensitivity for the detection of axillary lymph node metastases, whereas the PET/CT scan had higher specificity. Furthermore, ADC, SUV_max_ and SUV_max_ ratios showed some statistical significance among the patient groups according to different pathological parameters.

## Introduction

 In the female population, breast carcinoma (BC) is the most common type of cancer. 

 Mammography and ultrasonography are widely used in the detection of BC, but sometimes differentiating benign and malign breast lesions is quite difficult, especially in dense fibroglandular breasts. In order to be able to determine the operative treatment strategy of primary BC accurately, breast magnetic resonance imaging (MRI), in addition to X-rays mammography and ultrasonography, is substantially important (1,2). This modality does not use radioactivity and detects increased blood flow and tissue resolution in order to diagnose various types of cancer, thus, being more sensitive and accurate than mammography and ultrasonography (3). 

 Others reported that contrast-enhanced MRI has higher soft-tissue resolution and high sensitivity for BC (4, 5), yet still, it is difficult to differentiate between malign and benign breast lesions (6). Diffusion-weighted magnetic resonance imaging (dw-MRI) provides additional information about microstructural characteristics of tissue and is increasingly performed in evaluating tumours (7). Lately, some studies have shown that the apparent diffusion coefficient (ADC) value obtained from dw-MRI, as an indicator of water restriction in tumour, was useful in differentiating benign and malignant breast lesions, such as differentiating invasive ductal breast carcinoma (IDBC) from fibroadenoma (8, 9). 

 Recently, fluorine-18-fluorodeoxyglucose positron emission tomography and computed tomography (^18^F-FDG PET/CT) has been suggested in the staging of the BC, while the clinical examination is also important. The intensity of ^18^F-FDG uptake gives clinical and biological information about primary BC (10). 

 Some studies suggested that in BC the intense of ^18^F-FDG uptake is usually correlated with tumour size, high histological grade, Ki-67 index and the number of mitotic Figures (10, 11). 

 Several research papers have studied the diagnostic value of contrast-enhanced MRI or ^18^F-FDG PET/CT in BC lesions (12-15). Although both ^18^F-FDG PET and dw-MRI are useful for the detection of primary BC, there have been few publications comparing the diagnostic importance of maximal standardized uptake value (SUV_max_) and ADC acquired in the same primary and metastatic breast lesions before first surgery treatment (16-20). This study aimed that the diagnostic value of ^18^F-FDG PET/CT and dw-MRI was evaluated for the detection of breast tumour and axillary metastases of IDBC. ADC value from dw-MRI, SUV_max_ and rational SUV_max_, reflecting ^18^F-FDG uptake pattern of tumor in PET/CT scan, measured in same IDBC lesions, and also compared with clinicopathological parameters such as age, stage, axillary status, tumour histological grade, presence of lymphovascular and perineural invasion, and receptor status.

## Methods

***Patients***

 This is a retrospective study including the data of the patients admitted to our hospital between July 2010 and November 2013. The inclusion criteria were; a) diagnosis of IDBC, b) no previous breast surgery and neoadjuvant therapy c) having dw-MRI for diagnosis and ^18^F-FDG PET/CT scan for staging. One patient had two tumours, one in each breast, so 40 lesions were evaluated in 39 female patients with IDBC (mean age, 51.511.78 years; age range, 30-78 years). Prior to the imaging, breast cancer was diagnosed by a recent trocar needle biopsy in all patients. The ^18^F-FDG PET/CT and dw-MRI readers were blinded to the clinical history and previous examinations of the patients but were aware of the IDBC diagnosis. After imaging procedures, surgical intervention and ablation of the primary lesion were performed in all patients and also the ALN dissection and/or sentinel lymph node dissection and biopsy were performed in all patients. The mean time between PET/CT scan and surgery was 128.23 days. This study was approved by Non-invasive Clinical Research Ethics Committee of Bagcilar Training and Research Hospital (GOKAEK/ 2014-189). All procedures performed in this study involving human participants were under the ethical standards of the institutional research committee and with the 1964 Helsinki Declaration and its later amendments or comparable ethical standards. Due to the retrospective design of this study, the requirement for informed consent was not deemed necessary.

***The ***^18^***F-FDG PET/CT imaging protocol***

**Figure 1:**
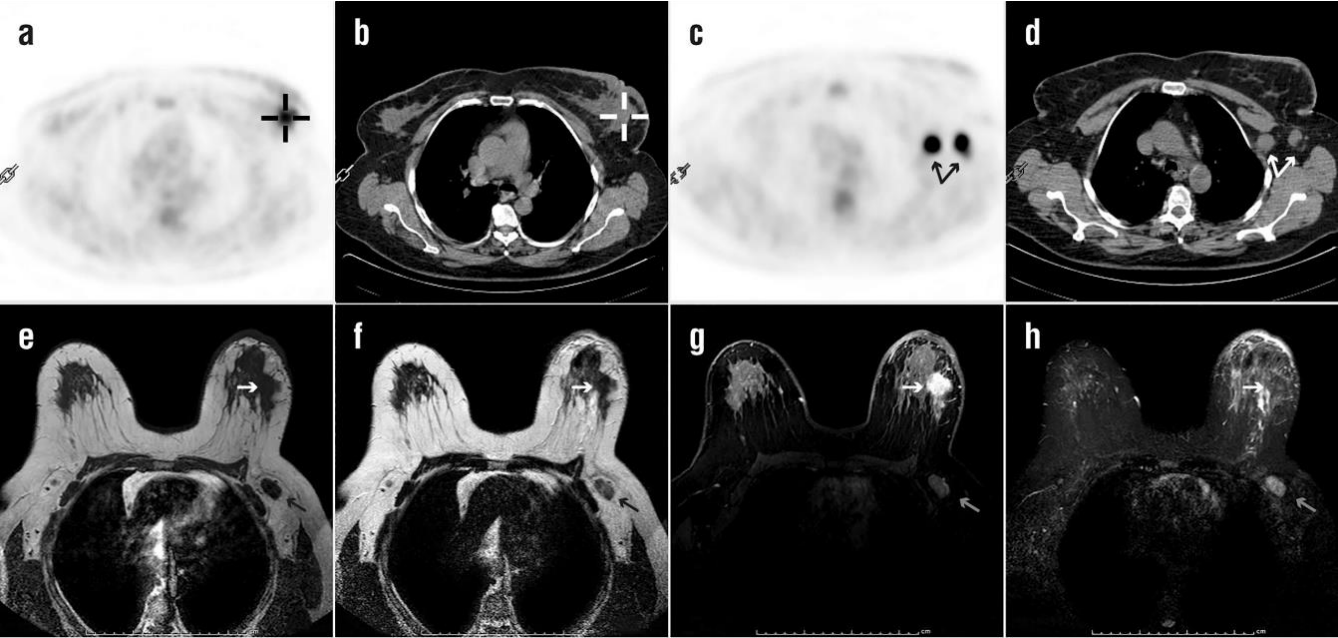
A 57 years old woman with IDBC of the left breast. On immunohistochemical study, ER and PR status were negative and Her2/*neu* was positive. Axial PET (**a**) and CT images (**b**) showed ^18^F-FDG uptake in the left breast signified by cursor at primary lesion with measured SUV_max_, 4.1. Axillary metastases with 8.9 SUV_max _were seen PET scan (**c**) and CT (**d**) images (**arrow**). The MRI lesion in the left breast (**white arrow**) had a hypointense signal with an irregular border at axial T1 (**e**) and T2 (**f**) weighted images. The lesion in dynamic sequences showed an increasingly homogenous contrast-enhanced mass (**g**), and in the short-time inversion recovery (STIR) sequences showed isosignal intensity mass (**h**). Axillary metastatic lymphadenopathy with decreased (or not) fatty hilus (**gray arrow**) was seen as iso-hypointense signal intensity in T1 and T2 weighted images with diffuse contrast-enhanced patterns at dynamic sequences. The calculated mean ADC value for primary lesion and axillary metastasis were 1.22×10^-3^ mm^2^/s and 0.95×10^-3^ mm^2^/s, respectively

**Figure 2:**
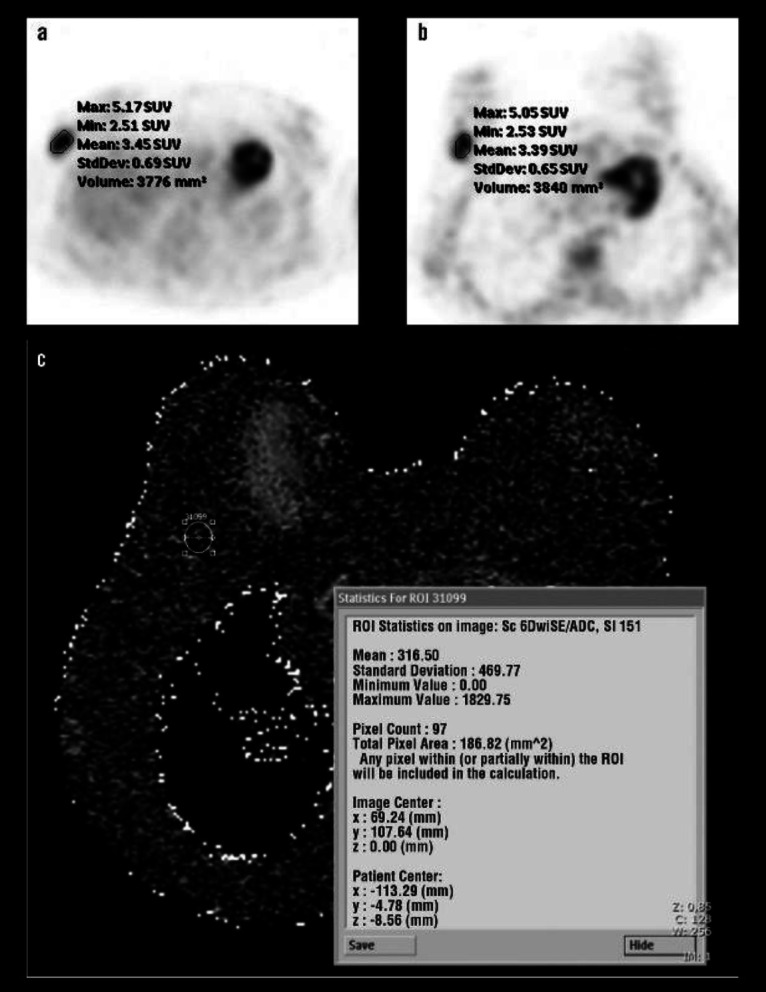
(**a**) Transaxial PET/CT images in supine position, (**b**) images at prone position obtained shortly after supine position images for accurate localization of primary tumour and (**c**) ADC map in diffusion-weighted imaging display, in a 40 year old woman with IDBC and 2.5 cm tumour size, grade 3, ER-PR positive, 1+ Her2/*neu* status in the right breast. ROIs are shown on (**a**) and (**b**) for measuring SUV_max_, and on (**c**) for measuring the mean ADC value

 The mean time between the trocar needle biopsy and PET/CT scan was 189.97 days. We thought that the biopsy did not affected the 18FDG uptake in primary tumour because of it was not detected 18FDG uptake in trace of needle and preoperative biopsy was small than 1 cm. For the PET/CT scan, patients were required to fast for 6h. Blood glucose levels were checked before injection of the radiopharmaceutical. The ^18^F-FDG PET/CT images were obtained by Gemini GXL PET/CT scanner (Philips Healthcare, The Netherlands). The dose of ^18^F-FDG was calculated as 2.5 MBq/kg body weight (±10%) and administered intravenously (i.v.) with a mean dose 379.2272.33 MBq and dose range 225.7-545.75 MBq in the veins or of the forearm or of the foot. During the waiting period after injection, patients rested in a quiet room. Scans from the vertex to the mid thighs were acquired approximately 1 h after the injection of ^18^F-FDG the patient lying supine with an empty urine bladder. Initially, for the CT part of the study, a contrast agent was given orally to all patients. The CT scan was acquired with the use of a standardized protocol involving 120kV, automatically calculated mAs for patients’ weight, a tube rotation time of 0.75s per rotation, a pitch of 0.85 and a section thickness of 3.3 mm. Instantly after the CT imaging, the PET scan was performed for 3 min per bed position and reconstructed using CT data for attenuation correction with iterative reconstruction. Whole body images were evaluated in transaxial, coronal and sagittal planes. The focuses of increased uptake in the primary lesions and the ALN lesions, if detected, were recorded (Figure 1). In addition, CT data were inspected for any findings in the above sites. The loss of fatty hilus and the increase of cortical thickening of the ALN suggested metastasis. The region of interest (ROI) was drawn over the hypermetabolic lesions and the maximum standardized uptake value (SUV_max_) was automatically calculated with polygonal free-hand ROI in all patients for all primary tumours (TmSUV_max_) and the ALN metastases (AxSUV_max_) (Figure 2). If there were multiple nodes with perceptible ^18^F-FDG uptake, the highest SUV_max_ was selected. For SUV_max_ ratio calculations, SUV_max_ was measured from the normal parenchyma of breast as background (bgSUV_max_) and liver (liverSUV_max_) with circular ROI. No tumour ROI was drawn larger than 1cm. For tumour-to-background SUV_max_ ratio calculation, SUV_max_ of the primary tumour, satellite lesion and metastatic ALN lesions, if detected with ^18^F-FDG PET/CT, was divided to background SUV_max_ as follows: 

a) For the primary tumour:

Tm/bg SUV_max_=TmSUV_max_ / bgSUV_max_

Tm/liver SUV_max_=TmSUV_max_ / liverSUV_max_

b) For the metastatic ALN lesions:

Ax/bg SUV_max_=AxSUV_max_ / bgSUV_max_

Ax/liver SUV_max_=AxSUV_max_ / liverSUV_max_

***The procedure of diffusion-weighted MRI imaging***

 The mean time between PET/CT scan and dw-MRI was 109.9 days. Dynamic contrast-enhanced breast MRI was performed with a 1.5 T MR scanner with multichannel capability (Philips Intera, Philips Healthcare, The Netherlands) using a standard four-channel phased-array breast coil in prone position. After acquiring a localizer sequence, an axial T2 weighted turbo spin-echo (TSE) sequence, a dynamic axial T1 3D fast low angle shot (flash) sequence by i.v. injection of gadodiamide (Omniscan, Amersham Health, Ireland), subtracted flash images, routine Short Tau Inversion Recovery (STIR) axial images and diffusion-weighted axial images with single-shot spin-echo echo-planar technique images were achieved including both breasts. The dw-MRI was acquired with motion-probing gradient (MPG) pulses applied along 3 (x, y, and z axes) directions with 3 different b factors, 0, 500, and 1000s/mm^2^. Maps of ADC were automatically generated on the operating console. Circular ROI was designated by one of the authors (Figure 2). Apparent necrotic or cystic components were avoided by referring to MRI images of other cases. The ADC from primary tumour (TmADC), was obtained automatically by measuring the intensity of the ROI on the ADC maps. Furthermore, if detected with dw-MRI, ADC values of axillary metastatic lymph node (AxADC) were also acquired.

***Pathology***

 Results of histopathology were used as the gold standard. Postoperational mastectomy specimens were analyzed by a breast pathologist and reported according to the College of American Pathologists protocol (21). 

 Modified Bloom Richardson grading system also known as Nottingham system was used (22). The components of this system consist of tubular structure, nuclear pleomorphism and number of mitoses, and each parameter is scored as 1 for the best and 3 for the worst. The sum score determines the “grade” ranging from I to III.

 In immunohistochemical analysis, estrogen (ER) and progesterone (PR) receptor status were scored as high (10%), low (1-9%) and negative (0%) (23). Values determined as low and high were accepted as positive. 

 Furthermore, Her-2/*neu* was classified with scores of 0, 1+, 2+ intense and 3+ based on the maximum staining intensity and stain distribution.

***Statistical Analysis***

 Statistical analysis was performed by NCSS (Number Cruncher Statistical System) 2007 Statistical Software (Utah, USA) in this study. All descriptive data were expressed as the sample mean ± the standard deviation (SD). The difference in SUV_max_ and ADC values of each clinicopathologic group, including age, T stage, American Joint Committee on Cancer (AJCC) stage, histologic grade, lymphovascular and perineural invasion, ALN status and receptor status, were analyzed to evaluate whether the SUV_max_ and ADC values can yield prognostic information of IDBC. In case the pathological groups were classified as positive-negative or yes-no groups, the Mann-Whitney U test and Kruskal-Wallis for variables with non-normal distribution were used. Values of P less than 0.05 were considered as significant. The One-Way analysis of variance (ANOVA) and Tukey’s HSD (honest significant difference) tests were used in groups with normal distribution. The correlation between ADC value and SUV_max_ was tested by Spearman’s correlation coefficient. 

 The sensitivity, specifity, positive predictive value (PPV), negative predictive value (NPV), and accuracy were calculated for dw-MRI and ^18^F-FDG PET/CT images for visual detection of the ALN metastases.

## Results

The mean tumour size was 2.420.99 cm (range 1.1-5 cm). The mean SUV_max_ and ADC for the primary lesions were 6.224.45 and 1.07310^-3 ^mm^2^/sn0.41910^-3^mm^2^/sn, respectively. The clinicopathologic features of our patients are listed in Table 1. 

 Primary lesions were detected in 39 (97.5%) of 40 lesions with PET/CT. The patient, in which lesion was missed by PET/CT, had 1.2 cm tumour size and histologic grade 2. The dw-MRI also detected primary lesions in 39 of 40 lesions. This patient, in which the IDBC lesion was not detected by dw-MRI, had a 2 cm tumour size with histologic grade 3, negative ER-PR and 3+ Her2/*neu* status.

 ALN metastases in 22 patients were detected by ALN dissection or sentinel lymph node biopsy. The sensitivity, specificity, PPV, NPV and accuracy of the ^18^F-FDG PET/CT scans for detection of metastatic lymph nodes in axilla were 40.9%, 88.9%, 81.8%, 55.2% and 62.5%, respectively. These values for dw-MRI were 40.9%, 83.3%, 75%, 53.6% and 60%, respectively.

**Table 1:** Clinicopathological characteristics of the patients

**Variables**	**n**	**%**
**Tumour size (according to TNM classification)**		
T1	18	45
T2	22	55
**Lymph node metastases**		
Negative	18	45
Positive	22	55
**AJCC Stage**		
1A	12	30
1B	1	2.5
2A	10	25
2B	8	20
3A	6	15
3C	3	7.5
**Histologic Grade**		
I	3	7.5
II	10	25
III	27	67.5
**Lymphatic invasion**		
Yes	26	65
No	14	35
**Vascular invasion**		
Yes	14	35
No	26	65
**Perineural invasion**		
Yes	15	37.5
No	22	55

AJCC: American Joint Committee on Cancer; IDBC: Invasive ductal breast carcinoma

 All SUV_max_ parameters and ADC values calculations were defined in the material and method section. There were no correlations between SUV_max_ parameters and ADC values for tumour and axillary metastasis (Table 2). 

 SUV_max _was measured from tumour (Tm

SUV_max_), the ALN metastases (AxSUV_max_), the normal breast parenchyma (bgSUV_max_) and liver parenchyma (liverSUV_max_), and SUV_max_ ratios were calculated for tumour-to-normal parenchyma such as Tm/bg SUV_max_, Tm/liver SUV_max_, Ax/ liver SUV_max_. 

**Table 2:** The correlation evaluation between SUV_max_ and ADC values

		**TmADC**	**AxADC**
TmSUV_max_	r	0.111	0.142
	P	0.500	0.910
Tm/bg SUV_max_	r	0.146	0.135
	P	0.374	0.914
Tm/liver SUV_max_	r	0.083	-0.191
	P	0.616	0.878
AxSUV_max_	r	0.050	-
	P	0.898	-
Ax/liver SUV_max_	r	0.050	-
	P	0.898	-

**r**: correlation coefficient; **P**: significance level (P value); TmSUV_max_: tumour SUV_max_; Tm/bg SUV_max_: tumour SUV_max_/normal breast parenchyma (as background) SUV_max_; Tm/liver SUV_max_: tumour SUV_max_/liver SUV_max_; Ax/liverSUV_max_: axillary lymph node metastasis SUV_max_/lung SUV_max_; TmADC: tumour ADC value; AxADC: axillary metastatic lymph node ADC value

 ADC, Tm SUV_max_ and SUV_max_ ratios were analyzed in each clinicopathologic group, including age, T stage, American Joint Committee on Cancer (AJCC) stage, histologic grade, lymphovascular and perineural invasion, ALN status and receptor status (Table 3). The patients with ER (p=0.007) and PR (p=0.036) positive status had lower mean ADC than the negative group. Tm SUV_max_ was lower in T1 than T2 tumour size (p=0.027) and PR positive patients than negative status (p=0.029). Tm/bg SUV_max_ was lower in PR-positive patients (p=0.004). Tm/liver SUV_max_ was higher in grade III patients than I and II (p=0.035) and PR negative status (p=0.043). There were no statistical differences for other clinico-pathologic parameters.

**Table 3:** The SUV_max_, SUV_max_ ratios and ADC values of primary tumour according to clinicopathologic parameters

**Characteristic**	**TmSUV**_max_		**Tm/bg SUV**_max_		**Tm/liver SUV**_max_		**TmADC (10** ^-3^ ** mm** ^2^ **/s)**	
	**Mean±SD**	**P**	**Mean±SD**	**P**	**Mean±SD**	**P**	**Mean±SD**	**P**
**Age**								
≤50 years	6.78±4.78	NS	5.73±4.04	NS	2.86±1.79	NS	1.01±0.42	NS
>50 years	5.57±4.07		6.16±5.12		2.33±1.64		1.13±0.41	
**T Classification**								
T1	4.79±3.77	0.027	5.02±3.10	NS	2.04±1.52	NS	1.12±0.56	NS
T2	7.33±4.71		6.63±5.33		3.07±1.77		1.03±0.26	
**AJCC Stage**								
1	4.81±4.19	NS	4.82±3.41	NS	2.70±2.10	NS	1.19±0.61	NS
2	6.97±5.22		6.86±5.54		3.89±2.77		1.02±0.29	
3	6.61±2.82		5.56±3.45		3.66±1.81		1.01±0.30	
**Grade**								
I	2.93±0.85	NS	4.30±0.97	NS	1.20±0.36	0.025	0.73±0.46	NS
II	4.27±3.16		3.27±1.74		1.79±1.27		0.99±0.40	
III	7.65±4.73		7.14±5.02		3.10±1.78		1.14±0.41	
**Nothingham prognostic index**								
Good	3.37±2.54	0.023	3.46±1.66	NS	1.46±1.16	0.015	0.88±0.46	NS
Moderate	7.43±5.05		7.09±5.21		3.04±1.88		1.18±0.41	
Poor	6.52±2.80		5.84±3.90		2.97±1.18		0.99±0.34	
**Lymphatic invasion**								
No	4.85±3.86	NS	4.73±3.38	NS	2.19±1.65	NS	1.12±0.35	NS
Yes	6.92±4.65		6.53±4.93		2.83±1.75		1.04±0.45	
**Vascular invasion**								
No	6.00±4.51	NS	5.82±4.16	NS	2.58±1.74	NS	1.08±0.32	NS
Yes	6.64±4.51		6.14±5.25		2.70±1.75		1.06±0.56	
**Perineural invasion**								
No	5.72±4.64	NS	6.24±5.57	NS	2.40±1.86	NS	1.07±0.46	NS
Yes	6.49±4.34		5.21±3.07		2.72±1.51		1.12±0.33	
**ALN metastases**								
No	5.79±4.74	NS	5.84±5.31	NS	2.45±1.88	NS	0.98±0.34	NS
Yes	6.56±4.31		5.99±3.92		2.75±1.63		1.17±0.48	
**ER status**								
Negative	8.36±4.19	NS	8.71±4.25	0.072	3.44±1.61	NS	1.42±0.51	0.007
Positive	5.70±4.24		5.32±4.25		2.44±1.72		0.98±0.34	
**PR status**								
Negative	8.36±4.19	0.029	8.81±5.71	0.004	3.41±1.72	0,043	1.26±0.44	0.036
Positive	5.16±4.27		4.49±2.99		2.23±1.62		0.97±0.38	
**Her-2/** ***neu***								
-	5.19±3.19	NS	5.57±4.31	NS	2.35±1.29	NS	1.34±0.55	NS
1+	6.26±5.18		4.96±3.66		2.48±1.93		0.99±0.35	
2+	4.60±3.37		7.78±3.84		2.14±1.31		0.82±0.49	
3+	7.60±4.90		6.67±5.8		3.16±1.98		1.03±0.28	

Tm: tumour; ADC: apparent diffusion coefficient; TmSUV_max_: tumour SUV_max_; Tm/bg SUV_max_: tumour SUV_max_/normal breast parenchyma (as background) SUV_max_; Tm/liver SUV_max_: tumour SUV_max_/liver SUV_max_; TmADC: tumour ADC value; SD: Stardard deviation; NS: Not significant; AJCC: American Joint Committee on Cancer; DCIS: Ductal carcinoma in situ; IDBC: Invasive ductal breast carcinoma; ER: Estrogen receptor; PR: Progesterone receptor

## Discussion

 There are many studies in the literature studying dw-MRI or ^18^F-FDG PET/CT which provide considerable functional information for the detection of BC, but few compare these two imaging modalities. There were some reports comparing the effectiveness of SUV_max_ and ADC values in detecting BC in the same study (16-20). In addition to the parameters evaluated in previous studies, we also compared SUV_max_ ratios with ADC amounts in our study because SUV_max_ is affected by factors such as serum glucose level, injection quality, body mass to fat ratio, injected dose of radiopharmaceutical and time after injection. In this respect, this study is the first to compare the ratios of ADC and SUV_max_. In addition, ALN status was evaluated with these two modalities in patients and compared with clinicopathological parameters.

 Both ^18^F-FDG PET/CT and dw-MRI overlooked 1 of 40 breast lesions with 97.5% sensitivity. One primary lesion, which was suspected to existent in a patient who was suffering from IDBC, could not be detected by PET, because of the lesion’s volume, which was 1.2 cm. Dual time point ^18^F-FDG PET/CT imaging yielded diagnostic accuracy, especially in suspicious breast lesions (24). We performed late imaging in this patient for detection of tumour, but there was no increase and detectability of ^18^F-FDG uptake in tumour site. The dw-MRI did not detect the primary lesion in a patient with negative ER-PR and 3+ Her2/*neu* status IDBC. In some studies, with a large number of patients, the sensitivity in detection of primary BC by MRI was 93% and 98.5% (25,26), and for ^18^F-FDG PET/CT were: 89.6% and 93% (26, 27). In our study, we had similar findings for MRI, with a higher sensitivity than expected for PET/CT. This may be due to the tumour size, which in our study was more than 1cm and to the fact that our BC patients had a more unique tumour pathology as being IDBC.

 The whole-body PET devices have specifically a limited capability to characterize small lesions, and breast abnormalities that are demonstrated with these devices can be difficult to localize anatomically. Dedicated high-spatial-resolution positron emission mammography (PEM) with detectors specialized for imaging the breast seems to be a promising agent in breast imaging. In a study, which compares the performance of PEM and MRI in detection of known breast malignancies, PEM tended to better depict cancer when it was present, indicating 92.5% versus 89.1% displayed with MRI. However, there was no significant difference in the detection rate between the two imaging modalities. 

 Researchers suggest that PEM is an alternative technique for patients who cannot tolerate MRI and is less likely to prompt unnecessary biopsies with improved specificity (28). 

 The most important prognostic factor in BC is the ALN status (10). Axillary LN metastases in 22, more than half of our patients, were diagnosed with surgery dissection or sentinel lymph node biopsy. It has been reported that although ^18^F-FDG PET/CT has good specificity for the detection of ALN metastases, its sensitivity is limited especially in early-stage cases (29). Similarly, in our study, we found high specificity (88.9% and 83.3%) and poor sensitivity (40.9% and 40.9%) for ^18^F-FDG PET/CT and dw-MRI, respectively. We wanted also to evaluate the axillary findings of SUV_max_ and ADC according to clinicopathological parameters, but the number of data for these values was limited in our study.

 In a meta-analysis, researchers evaluated the diagnostic accuracy of PET, with or without CT, and MRI in the evaluation of ALN metastases in early-stage BC. They found that ultra-small superparamagnetic iron oxide contrast agent (USPIO)-enhanced MRI showed a higher sensitivity and specificity than gadolinium-enhanced MRI and also showed that PET and MRI have lower sensitivity and specificity than sentinel lymph node biopsy. The studies in this meta-analysis showed a significantly higher mean sensitivity for MRI than for PET, with USPIO-enhanced MRI yielding the highest sensitivity. Some of the limitations of this meta-analysis were the limited number of patients and the fact that PET was not directly compared with MRI. Besides, MRI studies have a relatively small sample size and differ in methods In addition, MRI studies are relatively small and differ in methods (30). Our study is one of the few studies that directly compare PET/CT with breast dw-MRI with dynamic contrast. According to our results, sensitivity was lower in these two modalities compared to other studies and the specificity of PET/CT was slightly superior. Although the ^18^F-FDG PET/CT is not used instead of the sentinel lymph node sampling, it can be useful in preventing unnecessary sentinel lymph node sampling in patients who have positive findings in PET/CT scan owing to their high specificity.

 Tumours with high cellularity usually have lower ADC value (31, 32). On the other hand, ^18^F-FDG uptake and cellularity in BC are positively correlated (33). Therefore, SUV_max_ and ADC were expected to be in an inversely proportional correlation. However, in a recent study where 128 patients were monitored, researchers found no correlation between the ADC mean and SUV_max_ (17). Others have reported an inverse ratio in the context of malignancy in a smaller number of patients (18). Still others recently reported a weak inverse correlation between ADC and SUV that was not statistically significant (16). In the literature, we did not find information about the correlation between ADC and SUX_max_ ratios. Our study was the first report in that respect. As we could not find any correlation between ADC and SUV, there was no correlation between SUV ratios and ADC for primary lesion and satellite lesion in our study. Various results have been reported in studies with different malignancies. A strong correlation between SUV_max _and ADC was found in primary cervical cancer, but no significant correlation between both values for lymph node metastases was reported (34). An inverse correlation was obtained in pancreatic adenocarcinomas (35). In non-small cell lung carcinoma, this correlation was found in lymph node metastases and stronger in patients with squamous cell carcinoma than in patients with adenocarcinoma (36). Researchers showed that are significant differences depending on primary tumour histology in peritoneal carcinoma lesions (37). This indicates that histological features and the origin of tumour can be affected by the correlation between both values. Besides, the researchers showed that tumour stage influences the correlation of diffusion restriction and ^18^F-FDG uptake in non-small cell lung carcinoma (36). This suggests that local necrosis of big-size tumour can affect the ADC value according to ROI placement in tumour at the ADC map, whereas glucose metabolism in viable tumour cells adjacent of necrosis remains high. 

 We found that mean ADC values for ER and PR overexpression were statistically correlated. In some studies, researchers reported the same findings (17, 38), while another study reported the opposite (18). Ludovini et al. (39) have clarified that the cause for the lower ADC values detected in the ER-positive group as compared to the ER-negative group was that ER blocked the angiogenetic pathway and reduces perfusion, which in turn affects the ADC value. Most ER-positive breast tumour are also PR-positive status. The statistical difference in PR status for ADC was explained before with this condition (38). Unlike our findings, some researchers for ADC found statistically correlations with ALN status (38, 18, 40), tumour size (40), Her2/*neu* status (17, 20), histological grade (18, 40) and vascular invasion (18). 

 The SUV_max _as an indicator of ^18^F-FDG uptake in various publications is positively related to numerous clinicopathologic parameters such as tumour size, ALN metastases, histologic grade, and expression levels of ER, PR, Her2/*neu*, and Ki-67 among others (12, 14, 17, 18, 20). A higher SUV_max_ is related to higher relapse and mortality rates (14, 19). Furthermore, the preoperatively detected high ^18^F-FDG uptake was related to poor prognosis (16, 18). We found a significantly low SUV_max_ level in patients who had less aggressive tumour pathology such as tumour size, ER and PR positive status. However, there were some discordance among the SUV_max_ ratios. In another study, researchers reported that the tumour-to-contralateral breast, tumour-to-liver, tumour-to-lung and tumour-to-mediastinum SUV_max_ ratios were correlated with histologic type, tumour size, histologic grade, pleomorphism, mitotic count, lymphatic invasion, tumour necrosis, ER expression, Ki-67 index, ALN metastases, menopause and triple negativity (12). 

 This study has some limitations: the retrospective nature of this study, the small number of patients included, and the inability to correlate histological prognostic parameters with actual clinical prognosis at follow-up. Multifocal tumour was only detected in a few numbers of patients, which might be the reason of irrelevant statistical evaluation. The results are from a certain group of patients and cannot be inferred for all breast tumors. The comparison of metastatic ALN SUV_max_ and ADC values with clinicopathological parameters could be made due to the small number of patients. Additional assessments that complement these limitations are necessary.

## Conclusion

 Primary BC lesion detection rates by PET/CT and dw-MRI were high and same in this study. For the detection of metastatic ALN, ^18^F-FDG PET/CT scan had higher specificity than dw-MRI scan, while their sensitivity was equal. 

 Furthermore, SUV_max_ and SUV_max_ ratios correlated with several pathological parameters and ADC values were high in patients with ER and PR negative status. SUV_max_ and SUV_max_ ratios were more correlated with pathological parameters than ADC values. Even though we found no strong correlation between ADC and SUV_max_ or SUV_max _ratios in our study, these two imaging modalities might play a supplementary role in locoregional detection of IDBC in addition to distant metastases.

## Conflict of interest

 The authors declare that there is no conflict of interest, financial or otherwise.
